# Overweight and Obesity in School Children of a Hill State in North India: Is the Dichotomy Urban-Rural or Socio-Economic? Results from a Cross-Sectional Survey

**DOI:** 10.1371/journal.pone.0156283

**Published:** 2016-05-26

**Authors:** Madhavi Bhargava, S. D. Kandpal, Pradeep Aggarwal, Hem Chandra Sati

**Affiliations:** 1 Department of Community Medicine, Yenepoya Medical College, Yenepoya University, Mangalore, Karnataka, India; 2 Department of Community Medicine, Himalayan Institute of Medical Sciences, SRH University, Dehradun, India; 3 All India Institute of Medical Sciences, New Delhi, India; Institute for Health & the Environment, UNITED STATES

## Abstract

**Introduction:**

Overweight and obesity are a public health problem in India not only in adults but also in children. The authors sought to estimate the prevalence of overweight and obesity in school-going children of 6–17 years of age and examine its demographic and dietary correlates in context of their urban-rural status and socio-economic status.

**Methods:**

In this cross-sectional survey height and weight were measured in 1266 school children in government and private schools of urban and rural areas. Dietary assessment was done using single day 24-hour dietary recall method. The data were analyzed using SPSS (IBM SPSS Statistics Version 19) and WHO AnthroPlus Software. Factorial ANOVA was used for testing interaction within and between subgroups for continuous variables and Chi-square test was used for categorical variables.

**Results:**

It was found that the overall prevalence of overweight was 15.6% of which 5.4% were obese, with maximum prevalence in boys attending urban private schools. The mean caloric intake in the study population with 24-hour dietary recall method was 1558.2 kilocalories (SD: 428 kilocalories).

**Conclusion:**

Overweight and obesity is a significant problem in school-going children. Higher socio-economic status continues to remain an important driver of this epidemic in the younger generation and affects demographic and dietary determinants of this problem.

## Introduction

World Health Organization (WHO) estimates that, in 2008, more than 1.4 billion people worldwide were overweight, of these over 200 million men and nearly 300 million women were obese. India and China jointly account for total of 15% of total obese populationof the world [1].The prevalence of overweight and obesity has increased in most parts of the world among children and adolescents also[2]. According to 2013 estimates of global burden of disease (GBD), the prevalence of overweight and obesity in boys in developed countries is 23.8% and that in girls is 22.6%[1].Mean Body Mass Index (BMI), overweight and obesity are increasing world-wide due to changes in diet and physical inactivity[3].At least 2.8 million people die each year as a result of obesity[4].India is experiencing an epidemiologic and nutritional transition with increasing prevalence of non-communicable diseases (NCDs)[5, 6].There are reports from Indian subcontinent of increasing prevalence of overweight among children and adolescents during last decade, with co-existing high prevalence of undernutrition [7, 8].In case of children in India, the studies are mainly localized to particular regions or subgroups and are not nationally representative. Also the methods used to measure the prevalence of overweight have been heterogeneous and at times applied in an ad hoc manner [9].A meta-analysis of studies from India, regarding childhood obesity estimates prevalence of overweight as 12.64 (95% CI 8.48–16.8%) and that of obesity to be 3.39 (95% CI 2.58–4.21%)[10].With respect to Uttarakhand, a study in adult population from the state has reported prevalence of obesity as 13.9% in females and 11.1% in males [11]. Present study is a pioneer study for overweight and obesity in school children of Dehradun district of Uttarakhand, a hill state of North India using standard WHO cut-offs. It was done with the objective to estimate the prevalence of overweight and obesity in the school-going children (6–17 years age) and to assess the demographic and dietary correlates of overweight and obesity in this age group in context with their urban-rural and socio-economic status.

## Materials and Methods

### Study design

**Fig 1:**
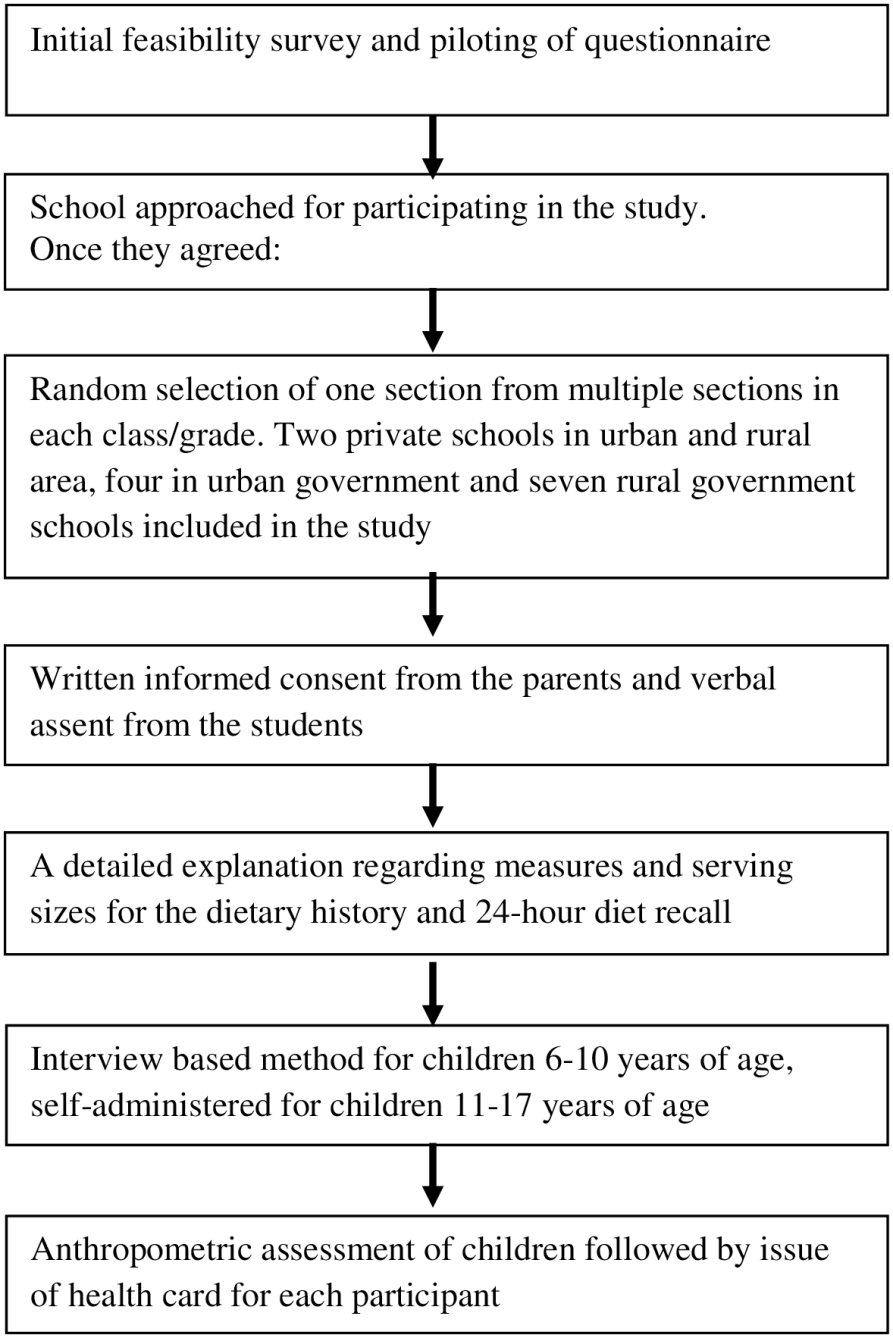
Outline of the study Process.

This was an observational cross-sectional survey conducted between June 2013 and May 2014 in Dehradun district of Uttarakhand state in North India.The outline of the study process is described in Fig 1.

### Pilot survey

An initial feasibility survey and piloting of questionnaire was done in schools in four settings; government and private schools in urban and rural areas. A formal letter describing research aim, background and relevance was explained. Following are some important observations made during this feasibility survey:

1. There were many private schools in the urban as well as the rural areas which were preferred mostly by the upper socioeconomic class.
2. The expenditure in terms of fees was very high in private schools and nominal in government schools.
3. Each grade had multiple sections, ranging from 3 to 5 in number.
4. The composition in a classroom, in terms of number of students and gender varied significantly in government and private schools. The number of girls was more in government schools throughout all grades and the number of boys in private schools increased in higher grades.
5. Private schools were not uniformly willing to participate and spare time for the study.
6. In case of questions on dietary recall, there was a need for demonstration of standard servings and cup size for better quality information from children.

Keeping these observations in mind it was decided to approach a required number of schools to achieve the target sample size.

### School types

The urban-rural type of the school is determined by its geographical location. For socio-economic status of the students, the private schools represented the higher socio-economic status and government schools represented the lower socio-economic status. The private schools having fees equal to or more than 40 USD per month were approached. In contrast, the government schools have fee structure of 2–4 USD per month and provide free mid-day meal till 8^th^ Grade.

### Sampling strategy

One section from each grade, grade 1 to 12, was selected by simple random sampling. All students present in the section were included in the study. A written informed consent was taken from parents or guardians and verbal assent was taken from the students.

### Sample size

Considering the prevalence of overweight and obesity in a multi-centric study as 23.9% (WHO cut-offs), and allowable error as 10% of the prevalence, the required sample size was 1266 (using the formula n = 4pq/L^2^)[12]. Equal number of participants was enrolled in all four sub-groups of the schools, i.e., urban private, urban government, rural private and rural government. This required participation from two schools each in urban and rural private; four urban government and seven rural government schools (number of students in rural government schools at times is very small).

### Study tools

A structured, pretested questionnaire was used with sections on demography and dietary pattern. It was interview method in local language for children of 6–10 years of age, and self-administered for older children. The first section consisted of demographic information including family type, birth order of the participant, parents’ education and occupation. The second section had information about dietary pattern in terms of type of diet (vegetarian, non-vegetarian, ovo-vegetarian), breakfast, lunch in school, number of meals per day, snacking, skipping meals, eating out, eating with screen on, fruit intake and a 24-hour dietary recall. Total caloric intake was calculated using National Institute of Nutrition, India guidelines[13]. Initially a standard presentation was made about the method for 24-hour recall in the class. This was followed by a demonstration of standard measures of cups, serving spoons, bowls and glass. All the queries were addressed to avoid incomplete or inaccurate information.

Anthropometric assessment was done by weight, height and BMI. Weight was recorded after removing heavy warm clothing, belt and shoes using a digital weighing machine with accuracy of up-to 100 grams (Omron^®^ Digital Model: HN 286). The weighing machine was standardized using a known weight at monthly interval during the study period. Height was measured using a wall mounted portable Staturemeter (Seca 206) with an accuracy of 0.1 centimeter. The participants were made to stand without shoes with heels slightly separated and back of head, shoulder blades, buttocks and heels brought in contact with the wall and head so positioned that the child looked directly forwards in Frankfurt plane. WHO Growth Standards, 2007 were used for calculating height-for-age, weight-for-age and BMI-for-age z-scores (HAZ, WAZ and BAZ);WHO cut-offs were used for defining overweight and obesity in children [14].

Following participation, each child was issued a health card with anthropometric record and its interpretation for parents.

### Inclusion and exclusion criteria

All students of consenting parents in the age group 6 to 17 years of selected schools were included in the study. Students with obvious disability or systemic illness known to be associated with weight gain or weight loss were excluded. This information was taken from the class teachers. Those students who were absent on the day of survey also got excluded automatically. Age of the child was determined using the date of birth in the school admission register.

### Statistical analysis

The data were analyzed using SPSS (IBM SPSS Statistics Version 19) and WHO AnthroPlus Software [15]. Factorial ANOVA was used to test associations at three levels. At first level, the association of area (urban vs. rural) with mean 24-hour caloric intake was tested, next this was tested for type of school (private vs. government). Finally interaction between area and type of school was tested for association with caloric intake. Chi-square test was used for categorical variables.

The study was approved by Ethics Committee of Himalayan Institute of Medical Sciences, SRH University (No: 2014/94).

## Results

Demographic characteristics of study participants are described in Table 1. A total of 1410 school students were screened. Of these, complete information available from 1266 participants was included in the analysis. The rest were excluded due to incomplete information in the 24-hour diet recall or non-availability of accurate birth dates. Out of total 1266 participants, 632 were from urban (private: 312; government: 320) and 634 were from rural (317 each from private and government) schools. There were 616 boys and 650 girls. Majority of the students (69.7%) reported nuclear families, maximum being in urban area (private 74.7%; government 81.9%). Birth order of more than two was more common in rural school children; maximum (45.7%) being in government school children in rural area. More school children in private schools in urban and rural area had parents who were graduates. Skilled and unskilled workers sent their children to government schools and parents with high-paying occupations (doctors, lawyers, architects, business, etc) sent their children to private schools.

**Table 1:** Demographic characteristics for study participants.

DemographicCharacteristic	Urban (N = 632)		Rural (N = 634)	
	Pvtn = 312	Govtn = 320	Pvtn = 317	Govtn = 317
**Gender**				
Boys	165 (52.9)	146 (45.6)	165 (52.1)	140 (44.2)
Girls	147 (47.1)	174 (54.4)	152 (47.9)	177 (55.8)
**Family type**				
Nuclear	233 (74.7)	262 (81.9)	189 (59.6)	198 (62.5)
Joint	79 (25.3)	58 (18.1)	128 (40.4)	119 (37.5)
**Birth order**				
≤ 2^nd^	286 (91.7)	190 (59.4)	278 (87.7)	172 (54.3)
> 2^nd^	26 (8.3)	130 (40.6)	39 (12.3)	145 (45.7)
**Father’s education**				
< Graduate	41 (13.1)	306 (95.6)	91 (28.7)	294 (92.7)
≥ Graduate	271 (86.9)	14 (4.4)	226 (71.3)	23 (7.3)
**Father’s Occupation**				
Service/ job	115 (36.9)	35 (10.9)	113 (35.6)	33 (10.4)
Business	97 (31.1)	57 (17.8)	102 (32.2)	71 (22.4)
Professional	78 (25.0)	0 (0.0)	77 (24.3)	5 (1.6)
Skilled worker	7 (2.2)	84 (26.3)	8 (2.5)	82 (25.9)
Unskilled worker	15 (4.8)	144 (45.0)	17 (5.4)	82 (25.9)
**Mother’sEducation**				
< Graduate	68 (21.8)	318 (99.4)	115 (36.3)	309 (97.5)
≥Graduate	244 (78.2)	2 (0.6)	202 (63.7)	8 (2.5)
**Mother’s Occupation**				
House-wife	211 (67.6)	233 (72.8)	233 (73.5)	255 (80.4)
Working	101 (32.4)	87 (27.2)	84 (26.5)	62 (19.6)

Figures in parenthesis indicate percentages.

Table 2 shows the distribution of participants according to nutritional status using BMI for age z-score cut-offs given by WHO. Overall mean z-score of both genders was at -0.47. Mean z-scores of participants of rural government schools was the lowest in all four sub-groups, being –1.31. More than 15% of the participants (M: 18%; F: 13.1%) were above +1SD or overweight of which 5.4% (M: 6.3%; F: 4.3%) were obese or above +2SD. Maximum obese children were in urban private schools, 11.9% followed by rural private, 8.2%. There were 13.6% (M: 14%; F: 13.2) participants who were thin (less than -2SD). Students classified as thin (<-2SD) were maximum in rural government schools, 28.7% as compared to urban government (14.7%). The difference in proportion of overweight and obesity in girls and boys was significant. But on stratifying the participant by area and type of school, this difference was no longer significant.

**Table 2:** Nutritional status of participants according to gender in each sub-group.

Gendern		BAZ Mean z-sore (SD)	BAZ <-2SD/Thin (%)	BAZ >+1SDOver- weight* (%)	BAZ >+2SD(Obese) (%)
**All Participants** †					
Boys	616	-0.47 (1.50)	86 (14.0)	111 (18.0)	37 (6.3)
Girls	650	-0.47 (1.31)	86 (13.2)	86 (13.2)	28 (4.3)
Total	1266	-0.47 (1.40)	172 (13.6)	197 (15.6)	68 (5.4)
**Urban Private**					
Boys	165	0.33 (1.50)	08 (4.8)	59 (35.8)	21 (12.7)
Girls	147	0.38 (1.26)	03 (2.1)	44 (30.1)	16 (11.0)
Total	312	0.35 (1.39)	11 (3.5)	103 (33.0)	37 (11.9)
**Urban Government**					
Boys	146	-0.97 (1.11)	24 (16.6)	07 (4.8)	01 (0.7)
Girls	174	-0.70 (1.01)	23 (13.3)	07 (4.0)	01 (0.6)
Total	320	-0.83 (1.06)	47 (14.7)	14 (4.4)	02 (0.6)
**Rural Private**					
Boys	165	-0.08 (1.42)	11 (7.3)	39 (23.6)	15 (9.1)
Girls	152	-0.06 (1.33)	12 (7.9)	32 (20.5)	11 (7.3)
Total	317	-0.07 (1.38)	23 (7.3)	71 (22.4)	26 (8.2)
**Rural Government**					
Boys	140	-1.33 (1.27)	42 (30.0)	6 (4.3)	2 (1.4)
Girls	177	-1.30 (1.02)	49 (27.1)	3 (1.7)	0
Total	317	-1.31 (1.13)	91 (28.7)	9 (2.8)	2 (0.6)

Figures in parenthesis indicate percentages; Pvt = Private; Govt = Government; BAZ = BMI-for-age z-scores; SD = standard deviation

*Overweight here includes obese;

^†^Chi-square boys vs. girls: p<0.05

The association of key demographic correlates with nutritional status of the study participants is as shown in Table 3. It was found that maximum prevalence of overweight was in urban private schools (32.7%) followed by rural private schools (22.4%). The proportion of school children with obesity was 10.2% in private schools irrespective of the area, urban or rural. The difference in proportions of participants in various nutritional categories in urban vs. rural and government vs. private were found to be statistically significant. While gender, age group and mother’s occupation did not have significant association with the nutritional status, type of family and birth order had a strong association with overweight and obesity. There was a greater proportion of overweight and obesity in nuclear family (10.1%; 4.3%) and lower birth order (11.7%; 6.4%). Similarly parental education and father’s occupation that indicated higher income, such as being professional or being in regular job and business were associated with higher proportion of overweight and obesity in the children.

**Table 3:** Demographic correlates of overweight and obesity.

Demographic Correlate	Nutritional Status (According to BAZ)				Chi Square test
	Normal -2SD-+1SD	Thin<-2SD	Overweight>+1SD-+2SD	Obese>+2SD	
Area of school					
Urban	457 (72.3)	58 (9.2)	78 (12.3)	39 (6.2)	99.19(p<0.001)
Rural	440 (69.4)	114 (18.0)	51 (8.0)	29 (4.6)	
Type of school					
Private	421 (66.9)	34 (5.4)	110 (17.5)	64 (10.2)	89.03(p<0.001)
Government	476 (74.7)	138 (21.7)	19 (3.0)	4 (0.6)	
Gender					
Boys	419 (68.0)	86 (14.0)	74 (11.7)	37 (6.3)	6.18(p>0.05)
Girls	478 (73.5)	86 (13.2)	58 (8.8)	28 (4.5)	
**Age group**					
6–9 years	261 (71.3)	42 (11.5)	35 (9.6)	28 (7.7)	3.15(p>0.05)
9–12 years	223 (66.8)	51 (15.3)	40 (12.0)	20 (6.0)	
12–15 years	246 (74.3)	45 (13.6)	31 (9.4)	9 (2.7)	
15–17 years	167 (71.1)	34 (14.5)	23 (9.8)	11 (4.7)	
**Type of family**					
Nuclear	643 (72.9)	112 (12.7)	89 (10.1)	38 (4.3)	9.55 (p<0.05)
Joint	254 (66.1)	60 (15.6)	40 (10.4)	30 (7.8)	
**Birth order**					
First and second	654 (70.6)	105 (11.3)	108 (11.7)	59 (6.4)	25.81 (p<0.001)
Third or more	244 (71.8)	66 (19.4)	21 (6.2)	9 (2.6)	
**Father’s education**					
< Graduate	549 (75.0)	140 (19.1)	33 (4.5)	10 (1.4)	151.40 (p<0.001)
≥ Graduate	349 (65.4)	31 (5.8)	96 (18.0)	58 (10.9)	
**Father’s occupation**					
Professional	102 (63.8)	7 (4.4)	34 (21.3)	17 (10.6)	105.59 (p<0.001)
Service	207 (69.9)	25 (8.4)	38 (12.8)	26 (8.8)	
Business	226 (69.1)	43 (13.1)	40 (12.2)	18 (5.5)	
Skilled worker	134 (74.0)	39 (21.5)	5 (2.8)	3 (1.7)	
Unskilled & others	229 (75.8)	57 (18.9)	12 (4.0)	4 (1.3)	
**Mother’s education**					
< Graduate	593 (73.2)	151 (18.6)	46 (5.7)	20 (2.5)	125.70 (p<0.001)
≥ Graduate	305 (66.9)	20 (4.4)	83 (18.2)	48 (10.5)	
**Mother’s occupation**					
House-wife	664 (71.2)	133 (14.3)	92 (9.9)	43 (4.6)	5.41 (p>0.05)
Working mother	233 (69.8)	39 (11.7)	37 (11.1)	25 (7.5)	

Figures in parenthesis indicate percentages; BAZ = BMI-for-age Z-score; SD = standard deviation

As far as dietary correlates were concerned there was a significant association with the habit of eating out and overweight and obesity as seen in Table 4. The condition had no significant association with type of diet (vegetarian and non-vegetarian), breakfast intake in the morning, number of meals per day, snacking and skipping meals. Surprisingly, overweight and obesity increased with increase in frequency of fruit intake and this association was statistically significant. But on stratifying the participants by government and private schools, this association was no longer significant. Less school children of government schools who consumed mid-day meal were overweight or obese. The proportion of children from private school eating their personal lunch who were overweight (16.0%) and obese (8.9%) was much higher (p<0.001).

**Table 4:** Dietary correlates of overweight and obesity.

Dietary Correlates	Nutritional Status (According to BAZ)				Chi square test
	Normal-2SD-+1SD	Thin<2SD	Overweight>+1SD -+2SD	Obese>+2SD	
**Type of Diet**					
Vegetarian	613 (72.1)	111 13.1)	84 (9.9)	42 (4.9)	2.40(p>0.05)
Non-vegetarian	93 (68.9)	19 (14.1)	14 (10.4)	9 (6.7)	
Ovo-vegetarian	191 (68.0)	42 (14.9)	31 (11.0)	17 (6.0)	
**Routine breakfast intake**					
Yes	651 (71.9)	121(13.4)	94 (10.4)	39 (4.3)	7.41(p>0.05)
No	246 (68.1)	51 (14.1)	35 (9.7)	29 (8.0)	
**Type of lunch at school**					
Mid-day meal	297 (75.0)	85 (21.5)	11 (2.8)	3 (0.8)	104.04(p<0.001)
Personal lunch	395 (67.8)	43 (7.4)	93 (16.0)	52 (8.9)	
**Number of meals per day**					
≤ 2 meals	194 (72.7)	33 (12.4)	28 (10.5)	12 (4.5)	0.98(p>0.05)
> 2 meals	704 (70.5)	138(13.8)	101 (10.1)	56 (5.6)	
**Snacking during day**					
≤ 1 per day	547 (71.2)	104(13.5)	78 (10.2)	39 (5.1)	0.34(p>0.05)
> 1 per day	351 (70.5)	67 (13.5)	51 (10.2)	29 (5.8)	
**Habits of Skipping meals**					
Yes	361 (70.6)	77 (15.1)	46 (9.0)	27 (5.3)	2.58(p>0.05)
No	536 (71.0)	95 (12.6)	83 (11.0)	41 (5.4)	
**Hotelling or eating out**					
< 1/week	472 (75.6)	107 (17.1)	35 (5.6)	10 (1.6)	78.35(p<0.001)
1/week	289 (67.4)	47 (11.0)	58 (13.5)	35 (8.2)	
>1/week	137 (64.3)	17 (8.0)	36 (16.9)	23 (10.8)	
**Eating while watching television**					
Yes	708 (71.1)	126 (12.7)	104 (10.4)	58 (5.8)	5.11(p>0.05)
No	189 (70.0)	46 (17.0)	25 (9.3)	10 (3.7)	
**Frequency of fruit Intake**					
Never	90 (75.0)	21 (17.5)	5 (4.2)	4 (3.3)	53.68(p<0.001)
1-2/week	388 (72.8)	89 (16.7)	32 (6.0)	24 (4.5)	
3-7/week	276 (69.9)	47 (11.9)	49 (12.4)	23 (5.8)	
>7/week	143 (65.6)	15 (6.9)	43 (19.7)	17 (7.8)	

Figures in parenthesis indicate percentages; BAZ = BMI-for-age Z-score; SD = standard deviation

**Fig 2:**
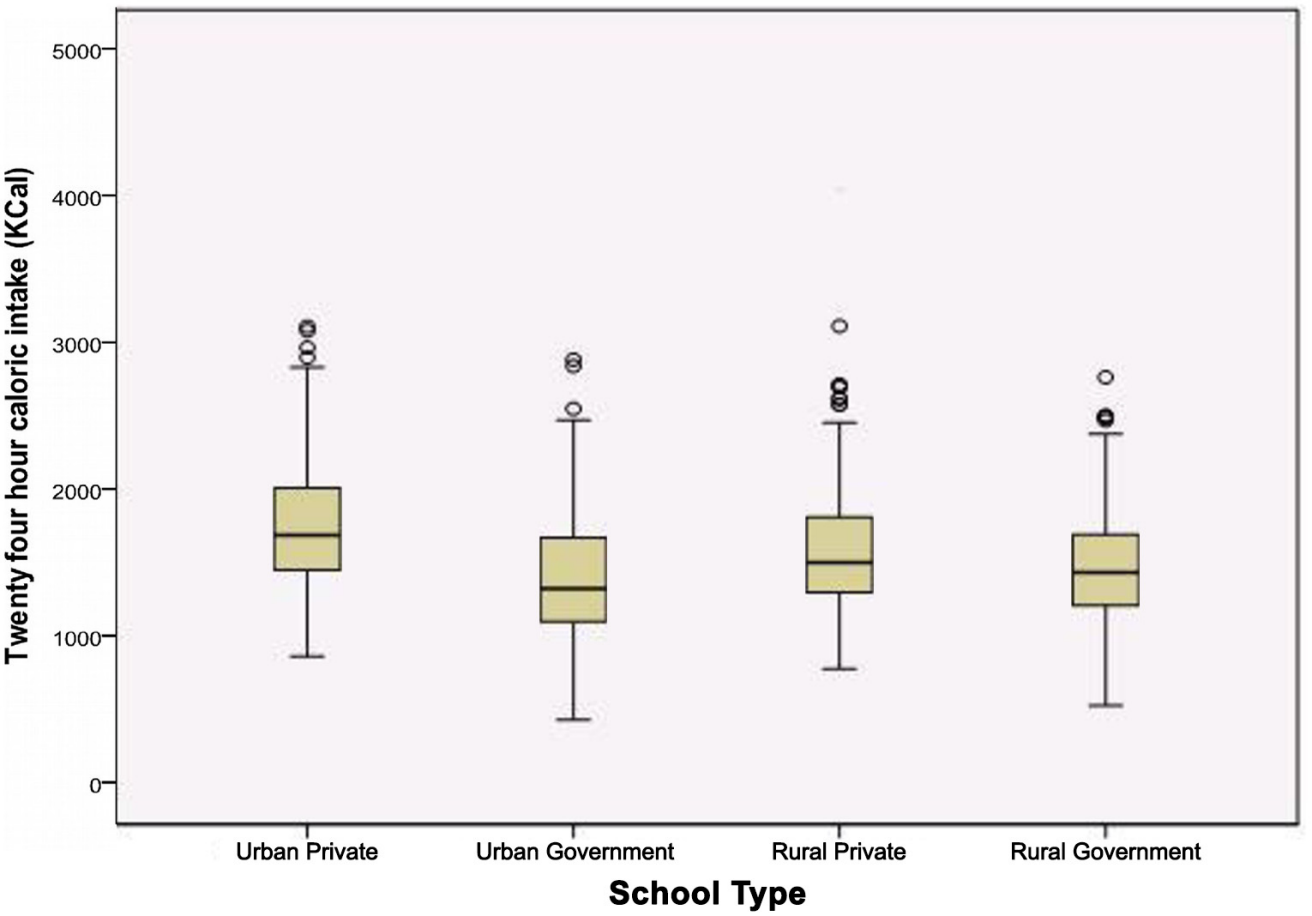
Caloric intake of participants of all four school subtypes.

**Fig 3:**
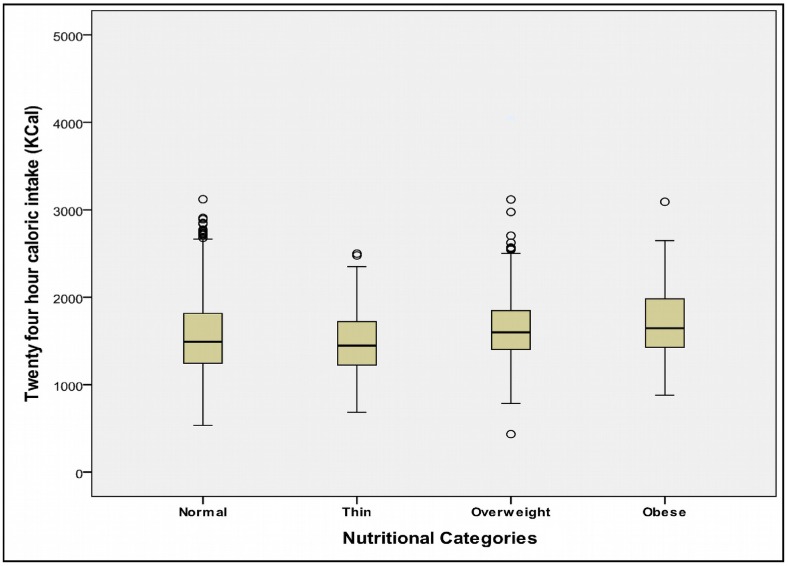
Caloric intake of participants of four nutritional categories.

A 24-hour caloric intake (Fig 2) in the entire subgroups and percent deficit when compared with recommended dietary allowance (RDA) for respective age groups is depicted in Table 5. The caloric intake of urban participants was higher than rural school participants; 1576.5 Kcal vs. 1539.8 Kcal. Maximum deficit was seen in urban government children where it was more than 30% of the RDA. Interestingly, as shown in Fig 3, the caloric intakes in obese children, though higher than other nutritional categories, the median is well below 2000 Kilo Calories.

**Table 5:** Twenty four hour dietary recall: Caloric intake and Recommended Dietary Allowance (RDA).

Subgroup	N(%)	Mean 24 hour caloric intake (Kcal)	Standard Deviation	^#^Percent Intake Deficit (Kcal)	Standard Deviation
Urban Private	312	1748.3	418.7	-13.9	19.7
Urban Government	320	1408.9	415.4	-30.7	19.8
**Urban Total**	**632**	**1576.5**	**450.0**	**-22.4**	**21.4**
Rural Private	317	1602.0	423.0	-20.5	21.6
Rural Government	317	1477.6	375.9	-26.3	21.1
**Rural Total**	**634**	**1539.8**	**404.6**	**-23.4**	**21.6**
**All participants**	**1266**	**1558.2**	**428.1**	**-23.0**	**21.5**

Kcal = Kilo Calories,

^#^ Deficit according to RDA for Indians

Factorial ANOVA was tested at three levels. At first level, the association of area (urban vs. rural) with mean 24-hour caloric intake was not found to be statistically significant (p = 0.091). Next the association between type of school (private vs. government) was found to be highly significant. Finally interaction between area and type of school was tested with post-hoc Bonferroni correction which was found to be significant (p<0.001).

As shown in Table 6, the ANOVA to test mean 24-hour caloric intakes between various nutritional categories, normal, thin, overweight and obese and it was found to be statistically significant (p<0.001).

**Table 6:** Association between mean 24 hour caloric intake (Kcal) and overweight/obesity.

Subgroup	Normal	Thin	Overweight	Obese	Mean Intake (SD)	Percent Deficit (SD)
**Urban Private**	1719.4(403.0)	1874.3(305.0)	1766.8(436.1)	1832.5 (489.0)	1748.3(418.7)	-13.9(19.7)
**Urban Govt**	1428.4(402.6)	1316.7(447.6)	1430.0(525.1)	925.3(63.6)	1408.9(415.4)	-30.7 (19.8)
**All Urban**	1554.5(427.5)	1422.5(476.1)	1715.0(463.6)	1785.9 (517.5)	1576.5(450.0)	-22.4(21.4)
**Rural Private**	1599.4(423.3)	1556.0(334.4)	1637.6(520.1)	1605.1(312.9)	1602.0 (423.0)	-20.5 (21.6)
**Rural Govt**	1470.3(387.6)	1480.4(346.8)	1633.1(442.6)	1588.5(75.6)	1477.6 (375.9)	-26.3 (21.1)
**All Rural**	1537.0(411.1)	1490.5(341.4)	1637.0(30.1.9)	1604.0(301.9)	1539.8(404.6)	-23.4(21.6)

Kcal = Kilocalories; SD = standard deviation; ANOVA (four nutritional categories with 24-hour caloric intake as dependent variable) F = 9.507; p<0.001

## Discussion

Overweight and obesity are usually multifactorial in origin. The present study found an overall prevalence of overweight as 15.6% of which 5.4% were obese. This prevalence of overweight is comparable to that of the range indicated by a review in India in 2007 (overweight: 8.5%–29% and obesity 1.5–7.4%) [16].A meta-analysis for childhood obesity studies done in India found overweight as 12.6% and obesity as 3.3% [10].The prevalence found was much lower than that in the two important multi-centric studies in the past; one by Khadilkar et al (23.9%) and another by Misra et al (18.5% of which 5.3% were obese) [11, 17].Both these studies have an advantage of being more representative due to multi-centric nature, but had participants representing urban India. Moreover, the Khadilkar study was done in urban schools catering to only the affluent children. Our prevalence of overweight and obesity seems to be higher in contrast to that recorded in National Family Health Survey—III (NFHS-III) which included age group 15–24 years. The prevalence of overweight was 3.5% in the age groups 15–24 in NFHS-III while overall prevalence across all ages was 15% in Uttarakhand [18].The latter is comparable to our findings. But it is important to note here that the cut-offs used by NFHS-III were that of adults (>25 kg/m^2^) even in the age group 15–19 years. This can obviously lead to underestimation of the problem of overweight in this age group.

The prevalence of overweight and obesity was found to be higher in boys as compared to girls in our study. Similar findings have been noted by various investigators [7, 19–21].On the contrary, findings of studies conducted by others indicate higher percentage of overweight and obesity in girls [22].In a study from South India, it was seen that prevalence of overweight was more in girls as compared to boys (9%; 5.9%); but that of obesity was higher in boys as compared to girls [23].The possible reason for lower prevalence of overweight and obesity in girls in our study could be due to high amount of physical activity in the form of household work and unequal distribution of food at household level.

The difference in prevalence of overweight and obesity in urban and rural school children was found to be statistically significant. Although there are individual studies that examine the prevalence in urban and rural areas separately, there are limited studies in Indian subcontinent comparing urban and rural prevalence. Most of these studies point towards greater prevalence of overweight and obesity in urban areas as compared to rural areas[22,24–25].In case of NFHS-III, the findings were similar although, it included only a section of age group (15–19) as compared to the present study. The prevalence of overweight and obesity in the, age-group 15–19 years was 23.5% in urban area and 6.2% in rural area[18].In a study in urban area of Karnataka, the prevalence of overweight and obesity was 13.2% and 6.8% in government and private schools [26]. Rural areas are primarily agrarian with physically active individuals who are usually seen as less vulnerable to overweight. But now there is rapid mixing of inhabitants of both areas and urban life style has penetrated rural communities as well. Retail shops and packed foods are a regular feature now in rural areas as well due to convenience and better shelf life.

The present study considered participants from private schools as belonging to upper socio-economic group and those from government schools as lower socio-economic group. Classification of socio-economic groups should be ideally done on the basis of family income. This was not possible in the present study as parents were not directly approached for the information. Out study found high prevalence of overweight and obese in urban private schools followed by rural private schools. On the other hand, this was found to be less in government schools of urban and rural areas. This method to represent socio-economic groups has been used by several school-based studies [27–30]. They have found significant difference in prevalence of overweight and obesity in children belonging to upper and lower socio-economic class. A study form Mumbai had high percentage of overweight children (22.9%) in private schools as compared to government schools. Education and occupation of the father make an important contribution towards economic status of the family. This in turn has probable implications on nutritional status of the children. Similar findings have been observed by Kolkata study [25].

The prevalence of overweight and obesity was higher in birth orders first and second and that of underweight was greater in higher birth orders. In a study in Odisha, no association was found between first vs. non-first child as far as overweight and obesity was considered [31].

Out of the various dietary correlates, the three correlates that have significant association with nutritional status were type of lunch at school (Government sponsored mid-day meal vs. personal lunch), habit of eating out and fruit intake. All these are also indirect indicators of economic status. Mid-day meal is a wholesome lunch provided by the state to all government school children till class 8. Due to expensive fruits, fruit intake in our study is simply a marker of affordability and shows positive association with overweight and obesity. The deficit in RDA is comparable in urban and rural areas, but the difference in deficit between private and government schools is very significant (30.7% in urban government and 26.3% in rural government). There is no published literature in India that looks into the deficit in RDA in terms of urban and rural residence and economic status in school children. But the rising food prices in India are a reality; they have obvious implications on caloric intake in some socio-economic classes. Very few studies are done in school children for their actual caloric intake. A study by Chaturvedi et al in 1996 found that in children 10–18 years of age, the caloric intake was deficient by 36%, 34% and 26% in 10–12, 13–15 and 16–18 years age groups when compared with RDA[32]. Studies that focus on caloric intake and its association with nutritional status are scarce in India. One study of 2005 found that mean caloric intake of children from 4–12 years of age who were overweight or obese was not significantly high, but the calories derived from fats was higher than desired by 25%. Recently, a Public Report on Health found that per capita energy consumption was 2379 Kcal per day [33].This was calculated using food diaries for the whole family, with dry weighment method. Also studies are required to examine the contribution of calories by unhealthy foods in children’s 24-hour diet and its effect on nutritional status

Strengths: Accurate anthropometric measurements were done by standard techniques by single observer which reduces the chances of inter-observer variation. This is a unique study in the region which includes urban as well as rural area with representation from private and government school for wider socio-economic coverage. Use of standard cut-offs lends the information collected for a valuable comparison for future large scale studies.

The prevalence of thinness is very significant in the study population. This indicates a double burden of undernutrition and overnutrition which is typical of rapidly developing economies with resultant income disparities [34].

Limitations: Cross-sectional nature in a limited region warrants caution before generalizing for a wider population of the state. A single day 24-hour recall was used for dietary assessment keeping in mind the younger children and their ability to recall. This ideally should have been an average of at least three days. Also the possibility of a recall bias cannot be ruled out. Moreover, the schools were not randomly selected and the participants are from a single district leading to selection bias and limited external validity.

## Conclusion

The present study found a significant prevalence of overweight and obesity in school-going children which co-exists with undernutrition. Affluence was found to be an important factor influencing the problem, irrespective of the urban-rural area. Higher socio-economic status affected many demographic and dietary correlates, irrespective of urban-rural area. School curriculum that includes education about diet as modifiable risk factor can address both ends of spectrum of malnutrition.
